# Success factors of a collaborative project to reduce healthcare-associated infections in intensive care units in Northeastern Brazil

**DOI:** 10.5935/0103-507X.20220070-en

**Published:** 2022

**Authors:** Ladjane Santos Wolmer de Melo, Thaís Moreira Estevão, Juliana Sousa de Castro Chaves, Janaina Maria Silva Vieira, Marialba de Morais Siqueira, Iêda Ludmer Guedes Alcoforado, Cláudia Fernanda de Lacerda Vidal, Heloisa Ramos Lacerda

**Affiliations:** 1 Hospital das Clínicas, Universidade Federal de Pernambuco - Recife (PE), Brazil.; 2 Hospital Universitário Oswaldo Cruz, Universidade de Pernambuco - Recife (PE), Brazil.; 3 Pronto-Socorro Cardiológico de Pernambuco, Universidade de Pernambuco - Recife (PE), Brazil.; 4 Hospital Metropolitano Oeste Pelópidas Silveira - Recife (PE), Brazil.; 5 Hospital Getúlio Vargas, Secretaria Estadual de Saúde de Pernambuco - Recife (PE), Brazil.; 6 Postgradute Program in Tropical Medicine, Universidade Federal de Pernambuco - Recife (PE), Brazil.

**Keywords:** Outcome assessment, health care, Outcome and process assessment, health care, Health evaluation, Quality improvement, Program development, Implementation science, Patient safety, Infections, Healthcare-associated pneumonia, Respiration, artificial, Intensive care units

## Abstract

**Objective:**

To describe the implementation and results of the collaborative PROADI-SUS project by the Brazilian Ministry of Health to reduce healthcare-associated infections: ventilator-associated pneumonia, primary central line-associated bloodstream infection and catheter-associated urinary tract infections.

**Methods:**

This was a prospective observational study that investigated the implementation stages and outcomes during 18 months in five intensive care units in the city of Recife. Reductions in healthcare-associated infections in each unit were calculated using previous medians compared to those of the study period.

**Results:**

The goal of reducing the three healthcare-associated infections, i.e., 30% in 18 months, was achieved in at least one of the healthcare-associated infections and was also achieved for two healthcare-associated infections in two hospitals and three healthcare-associated infections in just one hospital; the latter reached the target of 36 months. Implementing the bundles and monitoring the results by the professionals were considered essential actions by the local management teams. In addition, the acquisition of supplies and their availability alongside the beds, signage, checklists, staff awareness, adaptation, team building, training and celebration of achievements were assessed as being relevant for reducing healthcare-associated infections.

**Conclusion:**

The collaborative approach reduced healthcare-associated infections, despite partial adherence to the bundles. The hypothesis is that success is related to the project methodology and motivated multidisciplinary teams, especially nursing teams.

## INTRODUCTION

The Brazilian Ministry of Health, through the *Programa de Apoio ao Desenvolvimento Institucional do Sistema Único de Saúde* (Support Program for the Institutional Development of the Unified Health System) (known in Brazil as PROADI-SUS), promoted a collaborative project to reduce healthcare-associated infections (HAIs), called *Melhorando a Segurança do Paciente em Larga Escala no Brasil* (Improving Patient Safety on a Large Scale in Brazil),^(1)^ with a methodology called the “improvement model”, which is based on the Breakthrough Series (BTS) method from the Institute for Healthcare Improvement (IHI).^(2)^ Of the institutions that voluntarily applied, 120 adult intensive care units (ICUs) were selected to participate in the collaborative project. Of these, ICUs in five public tertiary hospitals, located in the Brazilian Northeastern Metropolitan Region of Recife, where approximately 4 million people live, were selected to participate in this study.

Collaborative projects are multifaceted organizational initiatives that unite professionals from various health departments or organizations in a collective effort to improve an aspect of care. They must contain five essential aspects: a specific topic to be addressed (when there is a large gap between knowledge and the common practice); clinical and quality improvement specialists; multidisciplinary teams from various locations; an improvement model (objectives, data collection and tests for change); and a series of structured activities (meetings and visits).^(3)^

Although collaborative studies are extensively used worldwide and have achieved a high percentage of effectiveness, few publications have described all aspects of the intervention and its components^(4)^ and exactly how the results were obtained.^(5)^ Moreover, because they are applied in different places and with different objectives, it is not possible to know whether effectiveness has depended on the chosen theme, or on local characteristics or teams. Therefore, comparing different teams within the same collaborative effort is recommended.^(6)^ More information regarding the factors that influence the outcome would mean that future collaborations could be adapted in order to increase their chances of success.^(7)^

According to the literature review, the five essential aspects of a collaborative (which are characteristics and factors of success^(3,6)^) and the taxonomies (which conceptualize the stages of implementation^(8)^ and its results^(9)^) of a collaborative improve the conceptual clarity , the relevance and the scope of the strategies,^(8)^ besides opening way for comparative studies.^(9)^

As a result of this PROADI-SUS project, a first article was initially published, which analyzed the quantitative performance of the five ICUs in Recife altogether and the indicators that made part of the study. This analysis was performed throughout the continuous assessment of the months studied.^(10)^

The objective of the present study however has been to describe the implementation and results of a collaborative project called PROADI-SUS implemented to reduce HAIs due to the use of devices and to identify factors that may have contributed to this reduction during the first 18 months of the national project in each of the five ICUs in Recife.

## METHODS

### From the collaborative project

In this collaborative study,^(1)^ the National Program for Patient Safety (PNSP - *Programa Nacional de Segurança do Paciente*) from the Ministry of Health defined the intended goals and selected the hospitals from the Unified Health System (SUS - *Sistema Único de Saúde*) to receive the interventions under the guidance and monitoring of the PROADI-SUS hospitals (HPS).

The HPS, also called centers of excellence, are certified as philanthropic, because they allocate part of their assistance to SUS, and are exempt from social security contributions.^(11)^ The change packages to be implemented and the indicators to be measured were defined by the HPS, Ministry of Health and IHI.^(1)^ Each of the five HPSs, called HUBs because they were centrally located as a reference for the implementation of the collaborative project, monitored 24 of the 120 participating hospitals. The five ICUs in Recife, which included 48 beds dedicated to the collaborative project, were linked to the same HUB. ‌

With the first face-to-face meeting, which included the local management team from all the hospitals, implementation of the collaborative project was initiated, whereby the main objective was to reduce the incidence densities (IDs) by 30% in 18 months and 50% in 36 months for the three main HAIs: ventilator-associated pneumonia (VAP), primary central line-associated bloodstream infection (CLABSI) and catheter-associated urinary tract infections (CAUTI). To achieve this goal, bundles were implemented to prevent HAI and increase adherence to basic hand hygiene protocols.

The hospitals agreed to participate in face-to-face learning sessions (FFLs; five during this period) and virtual learning sessions (VLs; monthly). They received educational visits each four months and virtual consultations by HPS facilitators.

The measures for HAI prevention and quality improvement were incorporated through PDSA (plan-do-study-act) rapid-cycle tests, in which the changes were first experienced with a small group of patients and health professionals. If the process was then considered successful and appropriate to the local reality, it was progressively implemented for the rest of the unit.

The infection IDs in 2017 (pre-project), the monthly IDs and data on meetings, reports, protocols, video classes, tools for testing change (PDSAs) and adherence to bundles were inserted into a single digital platform, thereby enabling the indicators to be monitored.

### From the study conducted‌

The methodology of the PROADI-SUS Project, which produced a robust database, enabled the development of several studies, such as the one in this article, which investigated important aspects of this type of intervention in each of the five ICUs. The indicators were calculated considering the period prior to the intervention compared to the study period.**‌**

In this prospective descriptive observational study, data were collected on a monthly basis for a period of 18 months, including a description of the success factors, the general characteristics of the hospitals, the stages of implementation and the results. The general characteristics of the hospitals described included the type of ICU and hospital, number of patients treated and patient days. The actions of implementing the collaborative project were reported by the Powell taxonomy:^(8)^ project financing and contract; definition of content and methods; development of educational and orientation materials; coordination of implementation; conducting face-to-face and virtual learning sessions; participation in face-to-face and virtual activities; building a coalition; guaranteeing resources; performance of tests and implementation of improvements; monitoring indicators; reports and sharing experiences; evaluation and feedback on the reports. The variables described included the activities of the local management team: monthly meetings, PDSAs performed and implemented and by type of infection, daily multidisciplinary rounds, extended daily visits by family members and in the unit with senior management (board), educational events, representatives present in the FFLs and VLs and reports from local management teams on factors that contributed to the success and difficulties encountered.

The results were described by Proctor’s taxonomy:^(9)^ acceptability, adoption, appropriateness, feasibility, fidelity, implementation cost, penetration and sustainability, and included the quantification of adherence to bundles (process indicators) and IDs (outcome indicators) to verify whether the goals were met in the first 18 months of the 36-month collaborative project.

### Definitions

The HAI surveillance was performed by professionals trained in infection control who had already been monitoring ICU patients before the project, using the definitions of the Centers for Disease Control and Prevention (CDC).^(12)^ In the case of ventilator-associated infection, the definitions of VAP were used. Their incidence was expressed as cases per thousand devices per day.

### Ethical aspects

The present study was authorized by the Ministry of Health and coordinated by the *Projeto Saúde em Nossas Mãos* (Health in Our Hands Project).^(1)^ and the participating hospitals. It was approved by the Ethics Committee of the *Hospital das Clínicas* at the *Universidade Federal de Pernambuco* (UFPE), under number 3,307,293.

### Assessment of the achievement of goals

The objectives related to the incidence densities of the HAI were a 30% reduction in the initial 18 months of the collaborative project, and a 50% reduction at the end of 36 months. For adherence to the preventive measures (bundles), the goal was 95% or more of execution. The bundles needed to be followed for each patient, and if any of the items were not met, they were considered as having been unfulfilled.^(13^-^15)^ These values and their percentage of variation before and during the interventions were calculated through the medians.

With regard to the characteristics of the work processes of the local management team, the goal was to hold meetings at least quarterly. For the other variables, since there was no target, the medians and percentages of the measured values were calculated to enable assessment of the hospitals.

The findings for each hospital were described independently. For some items, the information was the result of the 18-month assessment period, and for others in which there was monthly information, the median of the months and the total amplitude (minimum and maximum) of the period were calculated. In the assessments of the goals, the percentage was calculated considering the median of the period prior to the intervention in 2017 as a reference compared with the median of the subsequent period (2018 and 2019) using the formula:


 Goal % =(( Median of the year 2017 - Median of the year 2018/19)/ Median of the year 2017)×100

## RESULTS

Four ICUs were located in teaching hospitals, four were clinical-surgical and one was only clinical (H1, H here designates each of the five studied hospitals). Two ICUs were specialized: cardiac H1 (clinical) and neurological H3 (clinical-surgical) (Table 1). The work processes of the local management team in H1 and H5 occurred more frequently than in the others. The percentages of executing specific PDSAs for each infection were higher for VAP and BSI than for UTI and other subjects in all hospitals (Table 2).‌

**Table 1:** Characteristics of the five intensive care units studied and incidence densities of the infections related to healthcare in 2017 (pre-project)

Items	Hospitals				
	1	2	3	4	5
Characteristics of ICUs					
Clinical-surgical	No (clinical only)	Yes	Yes	Yes	Yes
Specialized	Yes (cardiac)	No	Yes (neurological)	No	No
Nursing dimensioning RDC 07/2010	Yes	Yes	Yes	Yes	Yes
Dimensioning of nursing Cofen	No	No	No	No	No
Teaching activity	Yes	Yes	No	Yes	Yes
Hospital beds > 400	No	Yes	No	Yes	Yes
Median patient days in the ICU > 300	Yes	Yes	Yes	No	No
Density of incidence of pre-project HAI					
Median ID VAP (min - max of monthly DI in 2017)	23.8 (12.9 - 62.5)	21.2 (11.1 - 70.2)	5.4 (0 - 21.5)	6.6 (0 - 16.4)	13.0 (7.7 - 35.5)
Median ID UTI (min - max of monthly DI in 2017)	13.6 (0 - 33.7)	9.9 (4.0 - 14.0)	2.1 (0 - 21.5)	0 (0 - 6.1)	0 (0 - 12.3)
Median ID BSI (min - max of monthly DI in 2017)	9.9 (0 - 20.2)	7.2 (3.6 - 11.1)	5.6 (0 - 22.2)	5.8 (0 - 12.6)	5.8 (0 - 15.4)

ICU - intensive care unit; RDC - Resolution of the Collegiate Board of Directors; Cofen - Federal Council of Nursing; HAI - health care-associated infections; ID - incidence density; VAP - ventilator-associated pneumonia; UTI - urinary tract infection; BSI - primary bloodstream infection.

**Table 2:** Work processes performed by the local management team in the 18 months of the study

Actions	Hospitals				
	1	2	3	4	5
Total meetings	68	29	44	33	67
Monthly meetings, median (min - max)	4 (2-5)	1 (0-5)	2 (0-9)	1 (0-4)	3 (1-7)
PDSAs performed (n)	63	18	67	58	51
PDSAs implanted (%)	66.7	72.2	25.4	29.3	72.5
ICU patients who received daily multidisciplinary rounds median %/month (min - max)	100 (100 - 100)	20 (18 - 39)	25 (12 - 74)	64 (34-67)	100 (100 - 100)
Duration in hours/day of extended family visit (hour)	12	0	9	2.5	10
Visits with senior hospital leaders (n)	17	3	1	9	7
Educational events for the team (n)	14	7	15	7	16
PDSA BSI (% of total PDSAs in 18 months)	23.8	27.8	19.4	32.8	9.8
PDSA UTI (% of total PDSAs in 18 months)	22.2	11.1	16.4	13.8	9.8
PDSA VAP (% of total PDSAs in 18 months)	36.5	27.8	38.8	15.5	41.2
Median number of hospital representatives in the VLs (n)	1	2	3	3	3

PDSA - plan-do-study-act; ICU - intensive care unit; BSI - primary bloodstream infection; UTI - urinary tract infection; VAP - ventilator-associated pneumonia; VL - virtual learning sessions.

The HAIs with the poorest pre-project medians were VAP and UTI in two ICUs and, in the others, VAP and BSI. The VAP represented the highest medians in all hospitals (Table 1).

**Figure 1:**
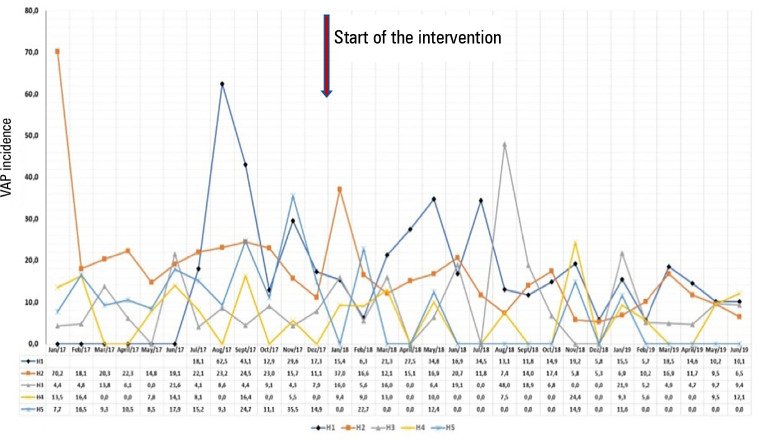
Incidence density of ventilator-associated pneumonia in the five intensive care units, from January 2017 to June 2019.

**Figure 2:**
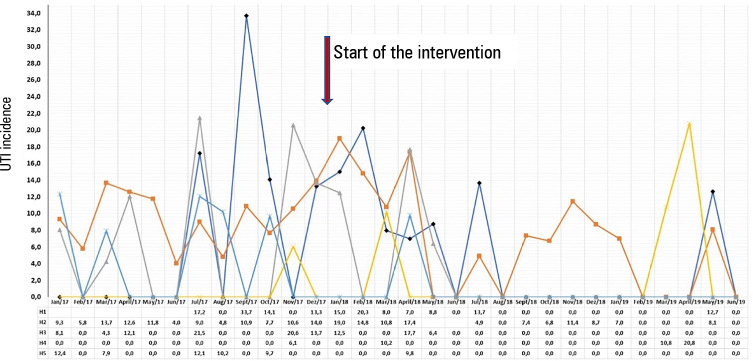
Incidence density of urinary tract infection in the five intensive care units, from January 2017 to June 2019.

**Figure 3:**
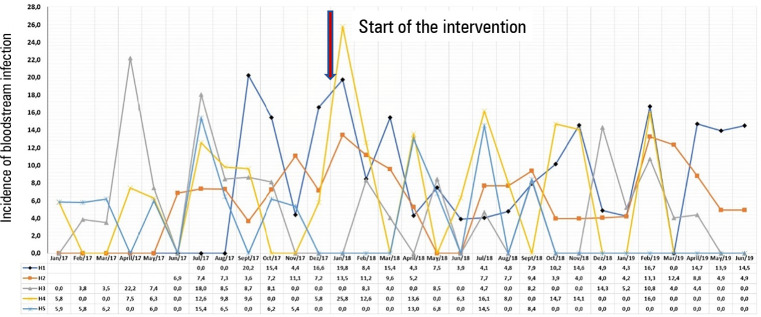
Incidence density of primary bloodstream infection in the five intensive care units, from January 2017 to June 2019.

Figures 1 to 3 demonstrate the variation of the IDs from the pre-project period until the end of the 18 months.

VAP - ventilator-associated pneumonia; H - hospital.

H - hospital.

The goal of HAI reduction was achieved in at least one of the infections in all ICUs. Two ICUs reached the target for two HAIs, and one ICU was successful in the three HAIs, and even reached the goals foreseen for 36 months (50% reduction). In two hospitals, in addition to meeting the goal in one of the HAIs, a second HAI decreased by 28% (Table 3). There was a reduction in the IDs during the study (Table 4).

**Table 3:** Goals achieved in reducing the median incidence densities of infections associated with healthcare and in adherence to prevention bundles, according to the median at the end of 18 months

	Hospitals				
	**1**	**2**	**3**	**4**	**5**
	**(%)**	**(%)**	**(%)**	**(%)**	**(%)**
Reduction of ID					
Reduced ID PAV	36	44	0	2	100^*^
Reduction ID UTI	100^*^	28	100^*^	100^*^	100^*^
Reduction ID BSI	17	0	28	45	100^*^
Adherence to bundles					
Bundle PAV	4.55	18.37	82.05	51.00	26.56
Bundle IDC insertion	50	100†	100†	33.33	100†
Bundle maintenance IDC	52.01	65.28	57.14	93.94	94.49
Bundle IDC insertion	61.9	67.00	88.50	76.39	86.94
CVC maintenance bundle	65.33	31.58	89.47	52.33	65.84

ID - incidence density; VAP - ventilator-associated pneumonia; UTI - urinary tract infection; BSI - primary bloodstream infection; IDC - indwelling bladder catheter; CVC - central venous catheter.

* Targets obtained for a 50% reduction of infections, planned for 36 months, which were already achieved in 18 months of the study. † Adherence goals of bundles obtained.

**Table 4:** Incidence densities before and during the project in the five hospitals over 18 months (medians)

Infections	2017	2018/2019
	(Before)	(During)
VAP	5.4 - 23.8	0 - 15.15
LCBSI	5.6 - 9.9	0 - 7.17
UTI	0 - 13.6	0 - 8.18

VAP - ventilator-associated pneumonia; LCBSI - laboratory-confirmed primary bloodstream infection; UTI - urinary tract infection

In terms of adherence to the bundles, the goals of the bundle including the insertion of an IDC were complied with in three hospitals (H2, H3 and H5). In the IDC maintenance bundle, H4 and H5 came very close to the target (93.94% and 94.49%). Most compliance (75%) was above 50% (Table 3).‌

The characteristics and success factors of the collaborative project were present in the five ICUs, as shown in table 1S (Supplementary material).^(3,6)^‌

The actions of the collaborative implementation were described by the Powell taxonomy, according to table 2S (Supplementary material). In the action “ensuring adequate resources”, H1 and H4 registered the acquisition of equipment, such as automatic beds, bedside support with alcohol gel, cuffometer, swabs with 70% alcohol, transparent film, signs to identify beds, goal setting and individual collectors for discarding urine. Investments in educational materials (leaflets, banners and adhesives) were described in all ICUs.

In the action “performance of tests and implementing improvements” to engage the ICU components, the local H1 management team motivated the employees and rewarded them with time off and gifts. H2 gave out awards to the team, and elected the professional of the month. H3 communicated the results to the team, seeking to educate them rather than punishing them. H4 held daytime and nighttime meetings, during which results were presented plus suggestions for the team to develop the PDSAs. In H5, a daily nurse and the formation of multidisciplinary teams to build the actions favored greater engagement.

The results, according to the Proctor taxonomy, are described in table 3S (Supplementary material). In relation to “appropriateness”, there was an initial expectation of the multidisciplinary team, especially nursing staff, that the project could lead to a greater demand for work, however, with the continuation of the project, it was considered compatible with routine care.

According to the local management teams, the collaborative project extended partnerships with sectors such as the Hospital Infection Control Commission (HICC) and Quality, in addition to a greater participation of ICU members, and the FFLs and VLs were positive. The experience of other hospitals encouraged the search for solutions using/adapting the strategies. Another positive point was the assistance of HPS tutors, adapting the proposals to the reality of the institution. The perseverance of the multidisciplinary leaders and the actions performed by the nursing staff were fundamental. Difficulty in engaging part of the medical team was reported during the 18 months observed.

The actions considered relevant by the local management teams to reduce HAI were: the implementation of bundles and following up the results by the professionals (indicated as essential by all); the acquisition of supplies and their availability alongside the beds (alcohol gel, cuffometer and hub scrub kit); signage (identifying dressings and equipment, marking the urine collection bag; signs indicating decubitus changes in order to prevent pressure sores; warnings for hand hygiene and “footprints” signaling the path to the sinks); checklist (central line insertion with the puncture kit); awareness of the team (presentation of the patient safety protocol and of the project to the team, before the changes are initiated; of the indicators in a wide view board); adaptation (using an angle meter to measure the inclination of the beds); creation of teams (to prevent each of the HAIs); training (on-duty shifts, with active learning methodologies and playful activities) and celebration of achievements (breakfast, incentive message or gifts). It should be noted that some actions were different for each institution.

## DISCUSSION

The strong point of this article is the description of the collaborative approach that enabled a quality improvement process in each ICU, culminating in the individual outcome of each unit.

With regard to reducing the HAIs, this goal was met in at least one outcome indicator in all ICUs. Two ICUs reached the target for two HAIs. One ICU was successful in the three HAIs, even reaching the goals set for 36 months. In two units, the goal (30% reduction) was met for one of the three HAIs, and for a second HAI, there was a decrease of 28%, i.e., very close to the goal. These findings are in agreement with the results found in a systematic review on the effectiveness of collaborative measures for quality improvement, based on compiled data from 1995 to 2014, in which 83% of studies conducted in hospitals demonstrated an improvement in at least one of the investigated indicators.^(4)^

The goal of adhering to bundles was obtained in three of the ICUs with the insertion of IDC and, in the other bundles, this was not achieved. Considering these five ICUs, the HAI reductions were mainly due to a decrease in the rates of using the devices, which were correlated with a reduction in the IDs of the VAP and UTI.^(10)^ Thus, because verifying the need to use the device and removing it as early as possible are two items of all bundles and are related to the reduction of infections,^(16)^ it is believed that compliance with these items may have contributed to the HAI reduction. It is understood that adherence to bundles, even below 95%, is able to reduce infections, as noted by Furuya et al. who demonstrated that, even with poor compliance with the bundle, when high adherence to one of the elements in the package was obtained, a reduction of 38% was estimated for the BSI.^(13)^‌

As this is an observational study, it was not possible to obtain a statistical estimate of the differences between the units, although hypotheses may be raised by observing the ICUs that presented different results.

It is believed that the HAI measurements were not underestimated, since in all hospitals there were trained professionals (nurses/doctors) responsible for ICU surveillance and for HAI diagnoses, using the same research methodology before and during the project.^(12)^ The teams appeared to change their behavior when care was improved for critically ill patients during the project. However, sustaining the improvement of the processes and results can only be confirmed through monitoring.

The description of the implementation of this collaborative project is important because it has the potential to generate information on what may or may not have contributed to the success of the approach,^(7)^ and it also presents aspects that have not been covered in previous studies^(4)^ (Table 2S - Supplementary material). The implementation occurred according to the original plan ^(1)^ and was depicted by the Powell taxonomy, used by Rohweder et al.^(17)^ Although we did not perform all the qualitative measurements of implementation by specific instruments, the implementation actions were assessed through observation, interviews and self-reporting, which are validated instruments.^(9)^

The five success factors^(3,6)^ were fully present. The topic chosen was appropriate because the frequency of HAIs associated with the use of devices is still a serious global problem,^(12)^ although there are well-validated prevention strategies.^(18^-^20)^ The experts in quality and in the chosen subject were the recognized technical teams of the Ministry of Health, HPS and IHI that used methods for knowledge acquisition and interaction between teams. The improvement method was BTS-IHI, one of the most widely used in the world and with good effectiveness.^(4)^ The choice of structured activities included meetings, face-to-face visits, virtual consultations, FFLs, VLs, monthly reports and training and/or motivational activities. The five determinants are not always present, as illustrated by a review of interventions for quality improvement, in which out of 175 projects, 58 (33%) did not meet these criteria. In the literature, the most frequently missed items are the fourth (having an improvement method) and the fifth (having structured activities).^(6)^ In a systematic review by Wells et al., of 1,095 selected articles, 848 (77%) were excluded because they did not meet all the criteria or did not present data on effectiveness.^(4)^ The presence of the five success factors may have contributed to the fulfillment of the proposals, as all the units reached the 18-month goal of a 30% reduction in the infection IDs for at least one infection.

The ICU with the best responses was in a teaching hospital that had a medical professional as the local project leader. The unit held almost monthly training sessions and/or motivational events (0.88). Furthermore, the hospital had already implemented and measured the prevention bundles for HAI through the HICC before the project, there was a nursing supervisor and leading member of the medical staff as active participants in the project, in addition to a trained multidisciplinary care team (nurses, nursing technicians, speech therapist, physiotherapists, doctors, nutritionist, psychologist, pharmacist), including nursing technicians graduated in nursing or other courses. A volunteer dental surgeon was also brought onto the team since the collaborative approach. A nurse and physician conducted multiprofessional visit daily. Most educational actions and PDSAs were initiated and/or prepared by ICU nurses. Despite the difficulty of compliance among professionals, within 18 months this ICU not only achieved the goal of a 30% reduction of the three HAIs, but also the intended reduction goal for the 36-month period of 50%. These findings are in agreement with those of Meredith et al.^(21)^ who observed how the composition of the team influenced the success of the collaborative approach. Donovan et al. indicated the interprofessional approach as being an essential component in providing high-quality care to critically ill patients since each professional category plays an important role in meeting the different needs of patients and relatives in the ICU.^(22)^

In the two ICUs with lower responses, there was a report of nonparticipation from the day-shift physician, a lack of supplies, a work overload of the local project leader, little involvement of the ICU medical team, absence of the day-shift nurse and insufficient hygiene professionals. Sometimes, efforts went unrecognized which therefore led to the discouragement of team members. The impaired work of the medical team may have contributed to lower responses, since Meredith et al.^(21)^ reported that working on changes with the medical team was positively related to the number of improvements, and the lack of physician participation was a significant barrier to collaborative implementation in California, US.^(23)^

In all hospitals, the persevering work of the teams, especially the nursing team, was outstanding, which is in agreement with studies that indicate these commitments as predictors of success in collaborative project to bring about quality improvement.^(6,7,24)^

## CONCLUSION

This study has demonstrated that the collaborative approach in five hospitals was effective in the five intensive care units in at least one outcome indicator, despite only partial adherence to the bundles. This may indicate that advances in adhering to the bundles, even below 95%, by improving the care, led to a reduction in infections in critically ill patients. The success factors of these intensive care units are possibly related to motivated professional teams in the various categories, especially nursing, and to the proposed collaborative methodology, including face-to-face and virtual meetings, tests of change and continuous monitoring.

## Figures and Tables

UTI - urinary tract infection; H - hospital.
