# Supplementary material for: Success factors of a collaborative project to reduce healthcare-associated infections in intensive care units in Northeastern Brazil
Source: Rev Bras Ter Intensiva. 2022 Jul-Sep;34(3):327–34. doi: 10.5935/0103-507X.20220070-en (PMC9749087; doi:10.5935/0103-507X.20220070-en)
Supplement: Supplementary file 1 [file rbti-34-03-0327-suppl01.pdf]

Success factors of a collaborative project to reduce healthcare-associated infections in intensive care units in Northeastern Brazil

Ladjane Santos Wolmer de Melo[1], Thaís Moreira Estevão[2], Juliana Sousa de Castro Chaves[3], Janaina Maria Silva Vieira[1], Marialba de Morais Siqueira[4], Iêda Ludmer Guedes Alcoforado[5], Cláudia Fernanda de Lacerda Vidal[1], Heloisa Ramos Lacerda[6]

Table 1S - Characteristics and success factors in the PROADI-SUS collaborative project in five Brazilian intensive care units, from January 2018 to June 2019, according to Schouten et al. and Hulscher et al.[3,6]

Determining factors[3,6]	Characteristics in the collaborative study
1. Selecting a specific topic where there is a large gap between knowledge and the common practice	1. Reduction of HAI associated with the use of devices in adult ICUs
2. Selecting quality and subject matter experts and their methods for transmitting best practices	2. Specialists in quality improvement, intensive care and infectious diseases of the Ministry of Health, PROADI-SUS Hospitals and IHI representatives applied varied didactics and, in most cases, with techniques that allowed the interaction between teams from different locations for the exchange of information and experiences. Plenary sessions with experts for the large group and workshops with discussions, reports or strategy development in small groups with participants from several different units.
3. Composing the collaborative group by several units and preparing teams motivated to participate	3. The composition of the studied group included the five ICUs participating in this study in the state of Pernambuco, which had local management teams motivated to participate.
4. Selecting the improvement method (objectives, collection and tests of changes)	4. The method was the "improvement model" based on the BTS-IHI
5. Choosing structured activities (activities, meetings, visits) to bring about changes and improvements	5. The choice of structured activities included meetings, face-to-face visits, virtual consultations, FFLs, VLs, monthly reports and training and/or motivational activities performed by the local management teams for the ICU teams.

HAI - healthcare-associated infections; ICU - intensive care units; IHI - Institute for Healthcare Improvement; BTS - Breakthrough Series; FFL - face-to-face learning session; VL - virtual learning session.

Table 2S - Implementation strategies of the PROADI-SUS collaborative project in five Brazilian intensive care units from January 2018 to June 2019, according to Powell taxonomy[8]

Strategies[8]	Description of the five participating ICUs
Funding and project contract<br>(*fund and contract for the clinical innovation*).<br>Governments and other service payers issue requests for proposals to deliver innovation, use contracting processes to motivate suppliers to deliver clinical innovation and develop new financing formulas that make it more likely that suppliers will deliver innovation[1]	The Brazilian Ministry of Health promoted and funded the collaborative project. The five institutions applied voluntarily, respecting the following criteria: a public or philanthropic, with more than 100 beds with 10 or more adult ICU beds; performing highly complex procedures; a regional reference center; with the potential for a positive response (institutional learning environment/safety climate) and a patient safety center. The adhesion contract was signed by the director of each institution. Through the PROADI-SUS, philanthropic hospitals (PROADI-SUS hospitals) (in Brazil, these are hospitals that allocate a percentage of their assistance to the SUS) were responsible for operationalizing the project with technical support from IHI and technical leadership from the National Program of Health Patient Safety/Ministry of Health
Definition of the contents and methods to be addressed<br>(*develop a formal implementation blueprint*).<br>Develop a formal implementation plan that includes all objectives and strategies. The plan should include the following:<br>- Objective/purpose of implementation<br>- Scope of change (e.g., which organizational units will be affected)<br>- Term and milestones<br>- Adequate performance/progress measures. Use and update of this plan is to guide the implementation effort over time)[2]	The Ministry of Health, PROADI-SUS and IHI hospitals validated the change packages and guiding diagrams for the reduction of the three HAIs associated with the use of devices - VAP, BSI and UTI - and for the Hand Hygiene Protocol proposed by the National Program of Health Patient Safety/Ministry of Health. Each activity was elaborated, agreed upon and validated.
Development of educational and orientation materials<br>(*develop educational materials*)<br>Develop and format manuals, toolkits and other support materials to make it easier for stakeholders to learn about innovation and for physicians to learn how to deliver clinical innovation[3]	The Ministry of Health and the PROADI-SUS Hospitals, in partnership with the IHI, were responsible for the development and preparation of content for the SAPs and SAVs, guiding diagrams, forms, tools for quality improvement and other face-to-face and distance learning strategies, promoting the interaction of different teams. Professionals from different units presented problems and solutions to other teams and everyone discussed the subject. The subjects of the scheduled SAPs and SAVs were also chosen according to the demand of the participating teams. In addition, the local hospital teams developed educational materials that they shared with one another.

Continue...

...continuation

Strategies[8]	Description of the five participating ICUs
Coordination of the implementation of improvement processes in participating hospitals (*create a learning collaborative*)<br>Facilitate the formation of provider groups or provider organizations and promote a collaborative learning environment to improve the implementation of clinical innovation[4]	PROADI-SUS, in which five Brazilian hospitals of excellence, also named PROADI-SUS Hospitals, applied their technical capacity and knowledge to develop safety practices. The specialists of the PROADI-SUS hospitals also guided the formation of local teams and distribution of well-defined roles of the components of these teams.
Conducting face-to-face and virtual learning sessions (*conduct educational meetings*)<br>Hold meetings targeting different stakeholder groups (e.g., suppliers, administrators, other organizational and community stakeholders, patient/consumer, and family stakeholders) to teach them about clinical innovation[5]	PROADI-SUS hospitals organized and conducted monthly VLs, lasting, in general, 1 hour, and five SAPs, with two days of immersion, in the same place where all were staying, and this allowed exchange of knowledge<br>The five ICU teams reported that knowing the experience of other hospitals with their difficulties and solutions stimulated them to also seek solutions to their problems, often using/adapting the strategies used by other institutions. The FFL always brought relevant topics for professional practice, in-depth knowledge on some subjects, and there was a climate of enthusiasm reported as being stimulating and contagious. In the VLs, members of the local management team participated, according to the programmatic content of the meeting. Both the FFLs and the VLs participated, at different times, in various professional categories (hospital director, physiotherapist, speech therapist, nurse, doctor), according to the learning objectives of the sessions. Other important learning moments were the face-to-face visits of the tutors of the PROADI-SUS hospitals to the participating ICUs and their virtual assistance. There were also local educational meetings directed to different teams from each hospital, such as HICC, Risk Management Unit, and senior management (management), technical services, nursing coordination, pharmacy and surgical center, to present the project and seek partnerships for the implementation of preventive measures for infections<br>Meetings with the families were rare but occurred in three units
Participation in face-to-face and virtual activities (*use in the implementation advisor*)<br>Seek guidance from implementation experts[6]	H1, H2, H3, H4, H5 participated in all VLs and FFLs and, on these occasions, received guidance from experts on the improvement model and implementation methods. In addition to these occasions, the instructions continued through the tutors of the PROADI-SUS hospitals, who were always available remotely and made periodic face-to-face visits. They tried to bring the proposals closer to the reality of each institution<br>The professionals of the units that directly received the teachings, participating in the FFLs and the VLs, were generally the components of the local project management team of each unit; however, these components varied according to the need to schedule the sessions, the availability of professionals and the initiative to include different members of the ICU or hospital of each unit in these sessions. As an example, on some occasions, the hospital board was present and other members of the HICC
Build a coalition<br>Recruit and cultivate relationships with partners in the implementation effort[7]	There were periodic meetings between the HUBs (which were the PROADI-SUS hospitals, central and reference points for the participating hospitals, and each HUB monitored 24 units in the project) with the objective of increasing the engagement in the implementation of the project, which occurred among the five participating hospitals and with the members of the care team from each unit of this study. The five ICUs in Recife were linked to the same HUB<br>The composition of the local management team of the project has already constituted the first partnership held within the hospital, as it consisted of professionals from the ICU and the Health Surveillance Service (containing HICC, Safety Center, Epidemiology Center and Quality Sector), which, from the beginning, enabled a greater interaction between the teams of these sectors. In addition, there was greater participation of members from the ICU multidisciplinary team, with the strengthening of this proposal, especially with the support of nursing team. There was also an expansion of the partnership with the hospital management, medical and nursing coordination. There were also reports of partnerships with nutrition and hemodialysis services to implement preventive measures for infections<br>Another important partnership included the tutors of PROADI-SUS hospitals who were always willing to assist and, during the various visits they made, always brought a lot of motivation<br>There were three local meetings between representatives of these five units, two held by local initiative and one coordinated by the PROADI-SUS hospitals responsible for these hospitals.

Continue...

... continuation

Strategies[8]	Description of the five participating ICUs
Ensuring adequate resources for the implementation of improvements (*change physical structure and equipment*)<br>Evaluate the current configurations and adapt, as necessary, the physical structure and/or equipment (for example, changing the layout of a room and adding equipment) to better accommodate the target innovation[8]	The directors of hospitals H1, H2, H3, H4, H5, through the commitment letter, agreed to develop at least 50% of the improvement actions foreseen in the change package, per semester. There was adaptation in the units through the acquisition of equipment or materials, according to local need and availability<br>In two units (H1 and H4), equipment such as automatic beds, bedside support with gel alcohol, cuffometer, swabs with 70% alcohol and transparent film were purchased. In another unit (H5), there was the inclusion of a volunteer dental surgeon working once a week in the ICU. In all ICUs, some investments were made in educational materials (leaflets, banners and adhesives)<br>In most units, there was no acquisition of equipment, improvement of the structure or hiring of human resources. In general, the improvements were due to changes in the processes
Performance of tests and implementation of improvements (*conduct cyclical small tests of change*)<br>Implement changes cyclically using small change tests before changing the entire system. Change tests benefit from systematic measurement, and the results of change tests are studied to gain *insights* on how to do better. This process continues in series over time, and refinement is added to each cycle[9]	H1, H2, H3, H4, H5 periodically performed cyclic change tests with improvements after each measured result (PDSAs) and, subsequently, implementations when the tests were effective and appropriate to the local reality<br>The development and execution of PDSAs improved over time, as the concepts were well understood after guidance from the tutors. There was an initial natural resistance to changes and the perception that these tests would generate a work overload, especially for nursing. This perception was overcome as the change processes resulted in improved results presented to the team (feedback). Nursing played a key role in the performance of tests and implementation of improvements.<br>In four of the five units, which are institutions linked to education, there were a large number of students (graduates and residents) who took turns monthly and a large multidisciplinary team. Therefore, it was necessary to pass the information several times, in the different shifts, as the test results were positive and needed to be expanded.
Monitoring of indicators (*conduct local needs assessment*)<br>Collect and analyze data related to the need for innovation[10]	H1, H2, H3, H4, H5 monitored the indicators presented in weekly rounds and monthly meetings and, according to the variations, received monthly guidance from the specialist tutors of the PROADI-SUS hospitals on the conduction of improvements<br>Data on adherence to bundles were collected by trained nurses, using a standardized form, and audited by an expert nurse of the HICC, with guidance for a minimum of 20 observations per indicator. The diagnosis of HAI was defined and confirmed by trained professionals and members of the HICC of the hospital, according to CDC criteria<br>The rounds with senior management occurred in all units with varying intervals
Referral of reports and sharing of experiences (*capture and share local knowledge*)<br>Capture local knowledge of implementation units about how implementers and physicians made something work in their environment and then share it with other locations[11]	H1, H2, H3, H4, H5 participated in 100% of the FFLs and VLs of the project. Nevertheless, during this period, there were three meetings with the five hospitals in Recife, in this study, also to share knowledge and experiences. The monthly reports, in addition to being discussed internally, were included in the digital platform, in which they were analyzed by PROADI-SUS hospitals for new orientations to the units<br>Four to five professionals participated in the FFLs, including sponsors and hospital leaders, in addition to other professionals from the local team, with alternation of categories, according to the guidelines of the PROADI-SUS hospitals, according to the programmatic content<br>In the VLs, there was always the participation of at least one member of the local management team, who then passed on the information discussed to the other members<br>As a way to support and exchange experiences, the five hospitals participating in the collaborative project in Recife held two meetings, in which the main difficulties were discussed and which strategies each service had used to overcome them. According to the reports, this experience was interesting because it allowed for greater learning and enabled exchanges between their teams. There was also a regional FFL, which occurred with these five participating hospitals, referred to as successful in exchanging experiences and that included the participation of family members of patients
Evaluation and feedback on periodic reports (*audit and provide feedback*)<br>Collect and summarize clinical performance data for a specified period of time and provide physicians and administrators to monitor, evaluate and modify the provider's behavior[12]	The local units collected the data and recorded them in a single platform from which the PROADI-SUS hospitals performed evaluations and were responsible for this analysis and feedback. The monthly report was prepared by professionals from the local management team and inserted in the digital platform. The data were monitored by the PROADI-SUS hospitals responsible person who evaluated and returned with guidance for planning new actions. The local leadership was responsible for disseminating the monthly results to senior management, multidisciplinary leadership and care professionals, in addition to making the results available in the unit's notice board for discussion in the local rounds.

ICU - intensive care unit; IHI - Institute for Healthcare Improvement; VAP - ventilator-associated pneumonia; BSI - primary bloodstream infection; UTI - urinary tract infection; FFL - face -to -face learning session; VL - virtual learning session; HICC - Hospital Infection Control Committee; H - hospital; CDC - Centers for Disease Control and Prevention; PDSA - *plan-do-study-act*; HAI - healthcare-related infections.

Table 3S - Description of the results of the collaborative implementation in five Brazilian intensive care units, from January 2018 to June 2019, described according to Proctor's taxonomy[9]

Concepts of implementation of results[9]	Implementation of the results in the collaborative project in the five hospitals of this study
**Acceptability**<br>Perception, among stakeholders in the implementation, that a particular treatment, service, practice or innovation is pleasant, palatable or satisfactory[13]	At the time of the initial presentation and detailing of the project to formalize the partnership, there was a perception by the local management teams of the five participating units that innovation would be satisfactory for improving the quality of care in the ICUs. After the local presentation of the project to the other participants (multidisciplinary team of each ICU and other sectors of the hospitals, such as pharmacy, social service and board of directors), there was a general impression that the project would be interesting if implemented in the ICUs
**Adoption**<br>Intention, initial decision or action to try or employ an innovation or evidence -based practice[14]	Adherence was obtained from the five ICUs of the selected hospitals that signed a contract with the hospital board in December 2017. The local teams decided, after knowing the project, to employ this evidence-based innovation in their ICUs. Representatives of each of the five hospitals participated during the following 18 months in 100% of the face -to -face and virtual meetings
**Appropriateness**<br>Perceived adequacy, relevance or compatibility of innovation or evidence -based practice for a particular practice environment, care team or consumer; and/or perception of the adequacy of the innovation to address a specific issue or problem[15]	The innovative method, through tests of changes in fast cycles, to ensure the implementation of preventive measures against HAI (bundles), was considered adequate because it is based on evidence. There was an initial expectation of the multidisciplinary team, especially nursing, that innovation could lead to a greater demand for work; however, with the continuation of the project, it was considered compatible with the care routine provided by the care team<br>From the follow-up of the project, it was clear to the teams that the collaborative strategy resulted in changes in care, observed by observing the daily work of the team because, in most hospitals, the completion and recording of the items of the prevention bundles of HAI were not part of the routine before the project
**Feasibility**<br>This is defined the extent to which a new treatment or an innovation can be successfully used or performed within a given agency or environment[9][16]	The innovation of seeking improvements through PDSAs for adherence to HAI prevention bundles was achieved by improving healthcare and significantly reducing infections in 18 months. There was an improvement in the knowledge of bundle items and the need to comply with them. However, adherence to all bundle items and their records were still insufficient because the goal of 95% was reached in three hospitals in three bundles. Even with adherence gaps, there was success, thereby proving the viability<br>All hospitals met the goal of reducing infection in 18 months in at least one of the HAIs. Two met in two, and one of the hospitals met the estimated goal for the three, including for 36 months
**Fidelity**<br>This is defined as the degree to which an intervention was implemented as prescribed in the original protocol or planned by the program developers[17]	The original project was fully implemented by the five hospitals, and there was also fidelity in the use of the main improvement tool, which were the PDSAs
**Implementation cost**	All planning and implementation costs were sponsored by the Brazilian Ministry of Health.
**Penetration**<br>This is defined as the integration of a practice within a service configuration and its subsystems[18]	All the multidisciplinary teams of the five ICUs were aware of the innovative way of testing changes (PDSAs) and the need to maintain preventive measures for HAI that were incorporated into daily practical routines<br>The PDSAs were used to test other ideas in the improvement of ICU care, different from those specific for reducing HAI. There was dissemination and use of the practice in other hospital ICUs, although not included in the original project.
**Sustainability**<br>This is defined as the extent to which a new implemented treatment is maintained or institutionalized within the stable, continuous operations of a service configuration[19]	Sustainability will be the subject of other studies, as the project continues for another 18 months, until December 2020

ICU - intensive care unit; HAI - healthcare-associated infections; PDSA - plan-do-study-act.

[1]Original text: "Governments and other payers of services issue requests for proposals to deliver the innovation, use contracting processes to motivate providers to deliver the clinical innovation, and develop new funding formulas that make it more likely that providers will deliver the innovation."[8]
[2]Original text: "Develop a formal implementation blueprint that includes all goals and strategies. The blueprint should include the following: 1) aim/purpose of the implementation; 2) scope of the change (e.g., what organizational units are affected); 3) time frame and milestones; and 4) appropriate performance/progress measures. Use and update this plan to guide the implementation effort over time."[8]
[3]Original text: "Develop and format manuals, toolkits, and other supporting materials in ways that make it easier for stakeholders to learn about the innovation and for clinicians to learn how to deliver the clinical innovation."[8]
[4]Original text: "Facilitate the formation of groups of providers or provider organizations and foster a collaborative learning environment to improve implementation of the clinical innovation."[8]
[5]Original text: "Hold meetings targeted toward different stakeholder groups (e.g., providers, administrators, other organizational stakeholders, and community, patient/consumer, and family stakeholders) to teach them about the clinical innovation."[8]
[6]Original text: "Seek guidance from experts in implementation."[8]
[7]Original text: "Recruit and cultivate relationships with partners in the implementation effort."[8]
[8]Original text: "Evaluate current configurations and adapt, as needed, the physical structure and/or equipment (e.g., changing the layout of a room, adding equipment) to best accommodate the targeted innovation."[8]
[9]Original text: "Implement changes in a cyclical fashion using small tests of change before taking changes system-wide. Tests of change benefit from systematic measurement, and results of the tests of change are studied for insights on how to do better. This process continues serially over time, and refinement is added with each cycle."[8]
[10]Original text: "Collect and analyze data related to the need for the innovation."[8]
[11]Original text: "Capture local knowledge from implementation sites on how implementers and clinicians made something work in their setting and then share it with other sites."[8]
[12]Original text: "Collect and summarize clinical performance data over a specified time period and give it to clinicians and administrators to monitor, evaluate, and modify provider behavior."[8]
[13]Original text: "The perception among implementation Stakeholders that a given treatment, service, practice, or innovation is agreeable, palatable, or satisfactory."[9]
[14]Original text: "Defined as the intention, initial decision, or action to try or employ an innovation or evidence-based practice."[9]
[15]Original text: "Perceived fit, relevance, or compatibility of the innovation or evidence-based practice for a given practice setting, provider, or consumer; and/or perceived fit of the innovation to address a particular issue or problem."[9]
[16]Original text: "It is defined as the extent to which a new treatment, or an innovation, can be successfully used or carried out within a given agency or setting."[9]
[17]Original text: "It is defined as the degree to which an intervention was implemented as it was prescribed in the original protocol as it was intended by the program developers."[9]
[18]Original text: "It is defined as the integration of a practice within a service setting and its subsystems."[9]
[19]Original text: "It has defined as the extent to which a newly implemented treatment is maintained or institutionalized within a service setting's ongoing, stable operations."[9]
